# Supplementary material for: Self-emergent vortex flow of microtubule and kinesin in cell-sized droplets under water/water phase separation
Source: Commun Chem. 2023 Apr 26;6:80. doi: 10.1038/s42004-023-00879-5 (PMC10133263; doi:10.1038/s42004-023-00879-5)
Supplement: Supplementary file 2 — Supplementary information [file 42004_2023_879_MOESM2_ESM.pdf]

Supplementary information for

Self-emergent vortex flow of microtubule and kinesin in cell-sized droplets under water/water phase separation

Hiroki Sakuta[1,2,†,‡], Naoki Nakatani[1], Takayuki Torisawa[3], Yutaka Sumino[4,*], Kanta Tsumoto[5], Kazuhiro Oiwa[6,7,*], Kenichi Yoshikawa[1,8]

[1] Faculty of Life and Medical Sciences, Doshisha University, Kyotanabe, Kyoto 610-0394 Japan

[2] Organization for Research Initiatives and Development, Doshisha University, Kyotanabe, Kyoto 610-0394 Japan

[3] Cell Architecture Laboratory, Structural Biology Center, National Institute of Genetics, Mishima, Shizuoka 411-8540, Japan

[4] Department of Applied Physics, WaTUS and DCIS, Tokyo University of Science, Katsushika, Tokyo 125-8585, Japan

[5] Division of Chemistry for Materials, Graduate School of Engineering, Mie University, Tsu, Mie 514-8507, Japan

[6] Advanced ICT Research Institute, National Institute of Information and Communications Technology, Kobe, Hyogo, 651-2492, Japan

[7] Department of Life Science, Graduate School of Science, University of Hyogo, Ako, Hyogo 678-1297, Japan

[8] Center for Integrative Medicine and Physics, Institute for Advanced Study, Kyoto University, Kyoto, Kyoto 606-8501 Japan

* Correspondence to: ysumino@rs.tus.ac.jp (Y.S.), oiwa@nict.go.jp (K.O.)

Present address

[†] Center for Complex Systems Biology, Universal Biology Institute, The University of Tokyo, Meguro, Tokyo 153-8902, Japan

[‡] Graduate School of Arts and Sciences, The University of Tokyo, Meguro, Tokyo 153-8902, Japan

Supplementary text

Reduction of mathematical model

For the simplicity, we use the equations (4)- (5) given in the main text with parameters, $\hat{\kappa} = 1$, $\widehat{D} = 0$, $\hat{\alpha} = 1$, and $\hat{\beta} = 1$. In this condition, $\bar{\zeta}$ is a bifurcation parameter, and the bifurcation point is $\bar{\zeta}_c = \kappa(1 + \alpha + \beta) = 3$. We here take $\bar{\zeta} = 3 + \varepsilon$. Noting that $\hat{\rho} = 1 + \delta\hat{\rho}$ and $\hat{\mu} = \delta\hat{\mu} + 1$,

$$\frac{\partial \delta\hat{\rho}}{\partial \hat{t}} = -\delta\hat{\rho} - \left[(3+\varepsilon)\frac{\partial^2 \delta\hat{\mu}}{\partial \theta^2} - \frac{\partial^2 \delta\hat{\rho}}{\partial \theta^2} + \frac{\partial^4 \delta\hat{\rho}}{\partial \theta^4}\right] - \delta\hat{\rho}\hat{v}, \tag{S1}$$

$$\frac{\partial \delta\hat{\mu}}{\partial \hat{t}} = -\delta\hat{\mu} - \left[(3+\varepsilon)\frac{\partial^2 \delta\hat{\mu}}{\partial \theta^2} - \frac{\partial^2 \delta\hat{\rho}}{\partial \theta^2} + \frac{\partial^4 \delta\hat{\rho}}{\partial \theta^4}\right] - \delta\hat{\mu}\hat{v}, \tag{S2}$$

with $\hat{v} = (3+\varepsilon)\frac{\partial \hat{\mu}}{\partial \theta} - \frac{\partial \hat{\rho}}{\partial \theta} + \frac{\partial^3 \hat{\rho}}{\partial \theta^3}$. We expand these equations using Fourier series expansion $\delta\hat{\rho} = \sum_{q=-\infty}^{\infty} \hat{\rho}_q e^{iq\theta}$ and $\delta\hat{\mu} = \sum_{q=-\infty}^{\infty} \hat{\mu}_q e^{iq\theta}$. Then, they lead to the expressions:

$$\begin{aligned}\frac{\partial \hat{\rho}_q}{\partial \hat{t}} &= -[1 + (1+q^2)q^2]\hat{\rho}_q + (3+\varepsilon)q^2\hat{\mu}_q \\ &+ \textstyle\sum_{q=q_1+q_2} \hat{\rho}_{q_1}\left[(3+\varepsilon)q_2^2\hat{\mu}_{q_2} - q_2^2(1+q_2^2)\hat{\rho}_{q_2}\right] \\ &+ \textstyle\sum_{q=q_1+q_2} q_1\hat{\rho}_{q_1}\left[(3+\varepsilon)q_2\hat{\mu}_{q_2} - q_2(1+q_2^2)\hat{\rho}_{q_2}\right],\end{aligned} \tag{S3}$$

$$\begin{aligned}\frac{\partial \hat{\mu}_q}{\partial \hat{t}} &= -(1+q^2)q^2\hat{\rho}_q + (-1 + (3+\varepsilon)q^2)\hat{\mu}_q \\ &+ \textstyle\sum_{q=q_1+q_2} \hat{\mu}_{q_1}\left[(3+\varepsilon)q_2^2\hat{\mu}_{q_2} - q_2^2(1+q_2^2)\hat{\rho}_{q_2}\right] \\ &+ \textstyle\sum_{q=q_1+q_2} q_1\hat{\mu}_{q_1}\left[(3+\varepsilon)q_2\hat{\mu}_{q_2} - q_2(1+q_2^2)\hat{\rho}_{q_2}\right].\end{aligned} \tag{S4}$$

Immediately, we find $\frac{\partial \hat{\rho}_0}{\partial \hat{t}} = -\hat{\rho}_0$,$\frac{\partial \hat{\mu}_0}{\partial \hat{t}} = -\hat{\mu}_0$ and right hand side of these equations do not have any higher order terms for $q = 0$. Thus, $q = 0$ modes converges to zero and is safely neglected in the following mode coupling terms. In short, the coupling of $q = 2$ and $q = -1$ contributes to $q = 1$ modes while the coupling of $q = 1$ terms contributes to $q = 2$ modes. To the lowest order, we need to consider,

$$\frac{\partial \hat{\rho}_1}{\partial \hat{t}} = -3\hat{\rho}_1 + 3\hat{\mu}_1 + \varepsilon\hat{\mu}_1 - 8\hat{\rho}_2\hat{\rho}_{-1} - 3\hat{\rho}_2\hat{\mu}_{-1} + 6\hat{\rho}_{-1}\hat{\mu}_2, \tag{S5}$$

$$\frac{\partial \hat{\mu}_1}{\partial \hat{t}} = -2\hat{\rho}_1 + 2\hat{\mu}_1 + \varepsilon\hat{\mu}_1 - 10\hat{\rho}_2\hat{\mu}_{-1} + 2\hat{\rho}_{-1}\hat{\mu}_2 + 3\hat{\mu}_2\hat{\mu}_{-1}, \tag{S6}$$

$$\frac{\partial \hat{\rho}_2}{\partial \hat{t}} = -21\hat{\rho}_2 + 12\hat{\mu}_2 - 4\hat{\rho}_1^2 + 6\hat{\rho}_1\hat{\mu}_1, \tag{S7}$$

$$\frac{\partial \hat{\mu}_2}{\partial \hat{t}} = -20\hat{\rho}_2 + 11\hat{\mu}_2 - 4\hat{\rho}_1\hat{\mu}_1 + 6\hat{\mu}_1^2. \tag{S8}$$

Bear in mind that $\hat{\rho}_{-q} = \hat{\rho}_q^*$ and $\hat{\mu}_{-q} = \hat{\mu}_q^*$. We introduce the combination of these variables to have eigen modes for the linear parts, $\phi_0 = -2\hat{\rho}_1 + 3\hat{\mu}_1$, $\phi_1 = \hat{\rho}_1 - \hat{\mu}_1$, $\phi_2 = \frac{-\hat{\rho}_1+\hat{\mu}_1}{2}$, and $\phi_3 = \frac{5\hat{\rho}_2-\hat{\mu}_2}{2}$. The corresponding eigenvalues are $\lambda_0 = 0$, $\lambda_1 = -1$, $\lambda_2 = -1$, and $\lambda_3 = -9$. For small ε and hence small ϕ_0, the modes ϕ_1, ϕ_2, and ϕ_3, quickly converge to the steady values described by slow mode ϕ_0. We find, $\phi_1 = 0 + O(\phi_0^4)$, $\phi_2 = 0 + O(\phi_0^3)$, and $\phi_3 = \frac{2}{9}\phi_0^2 + O(\phi_0^3)$. At this order, $\hat{\rho}_1 = \hat{\mu}_1 = \phi_0 + O(\phi_0^4)$, $\hat{\rho}_2 = \hat{\mu}_2 = \frac{2}{9}\phi_0^2 + O(\phi_0^3)$. Finally, we obtain,

$$\frac{\partial \phi_0}{\partial \hat{t}} = -\frac{10}{9}\phi_0(|\phi_0|^2 - \frac{9}{10}\varepsilon). \tag{S8}$$

The result shows that the type of observed bifurcation is super critical pitch-fork bifurcation, and the steady values of $|\phi_0|$ converges to $|\phi_0|_c = \sqrt{\frac{9}{10}\varepsilon}$ when $\varepsilon > 0$. The largest speed at the interface is shown from $\hat{v}_1 = i(1+\varepsilon)\phi_0$, and $\hat{v} \simeq \hat{v}_1 e^{i\theta} + (\mathrm{c.c})$. We find the maximum speed will be $2(1+\varepsilon)\sqrt{\frac{9}{10}\varepsilon}$, which is plotted in Fig. S3. This estimation shows good agreement with our numerical calculation also shown in supplementary figure S3.

Supplementary figures

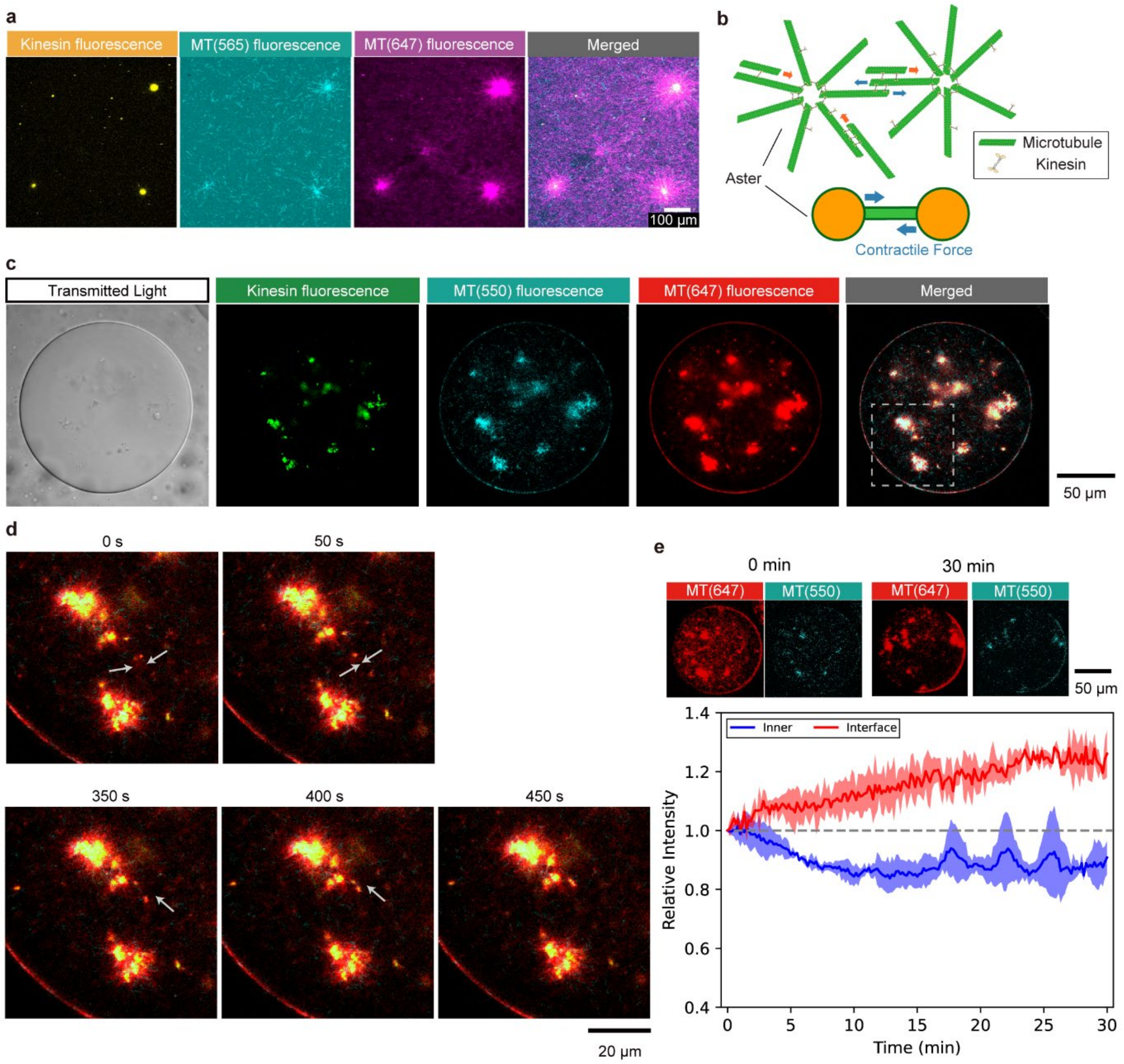

Supplementary Figure S1. Formation of an aster-like structure upon the combination of MT and 4-headed kinesin KIF5B$_{\text{head}}$-Eg5$_{\text{tail}}$. a, Formation of aster-like structure in the bulk solution without the presence of DEX and PEG (without w/wPS droplets) as confirmed in our previous study[39]. Kinesins were visualised using fused green fluorescent protein (eGFP). To visualise the MT detailed structure, two types of fluorescence-labelled MTs were prepared, ATTO565 (MT(565)) and ATTO647N (MT(647)). By mixing MT(565) and MT(640) at a ratio

of 1:10, MT(565) fluorescence shows typical aster-like structures: radially-extending MTs from a node where kinesin is concentrated. **b,** Schematic illustration of an aster-like structure and a dynamic contractile network originated from aster-kinesin complex as in centre of the illustration. **c,** Formation of aster-like structure in the w/wPS droplet in the presence of DEX and PEG. Two types of fluorescence-labelled MTs were mixed, MT(550) (ATTO550; labelling ratio 9.8%) and MT(647) (ATTO647N; labelling ratio 6%) at a ratio of 1:10. The mixture enabled us to observe individual MTs in the aster-like structures (See the panel of MT(550) fluorescence). **d,** Time-coarse images observing the size growth of aster-like structure. The several μm-sized aster-like structure aggregate with neighbouring one and become larger by repeating the aggregation upon the contractile network. **e,** Time-course of distribution of MTs at the interface and inner of droplet obtained by the fluorescent labelled MT (ATTO550 accounting for 1/10 of total MT). The plotted line represents the average values of three droplet and the band around line indicate the S.D..

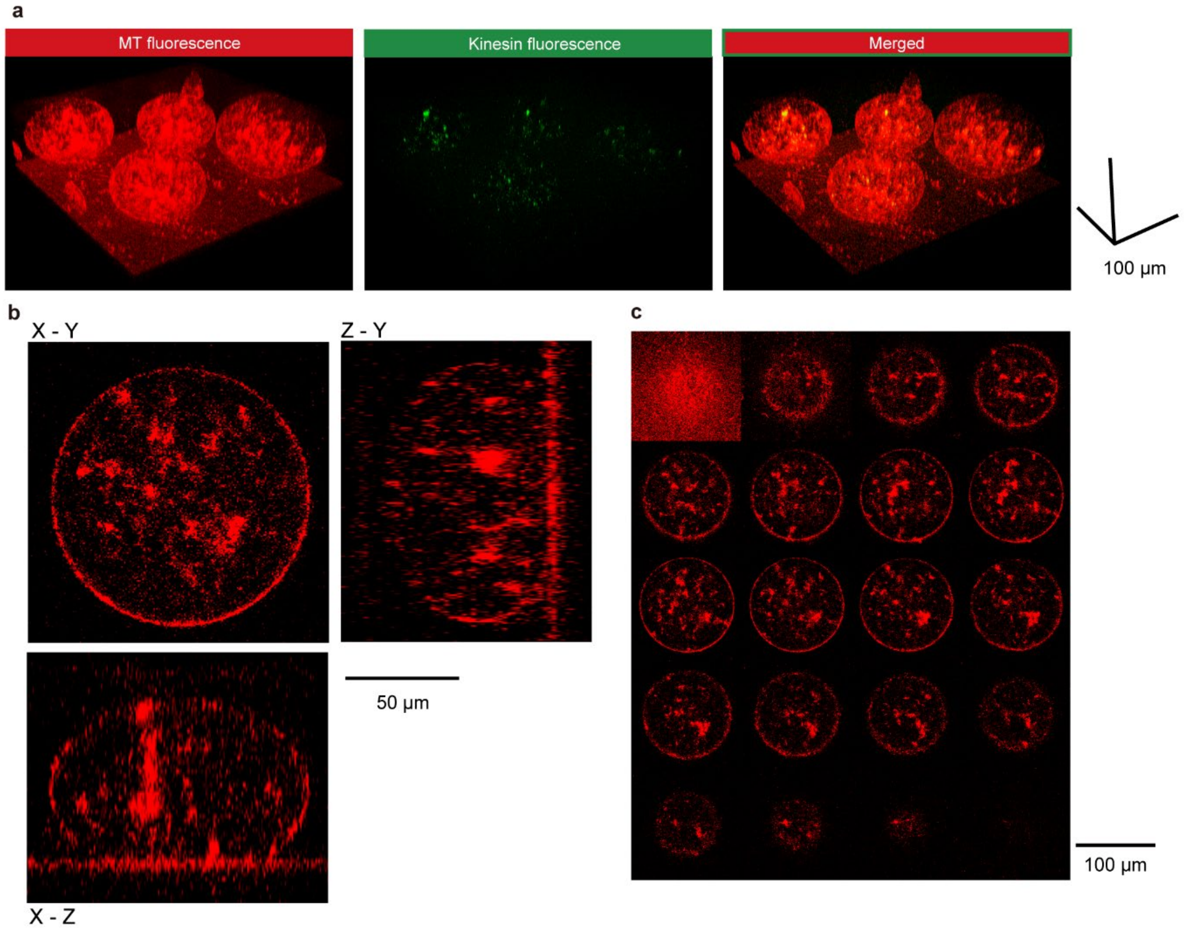

Supplementary Figure S2. 3D image of a droplet rendered from the confocal image along the *Z*-axis. a, 3D fluorescent image of several droplets. **b,** Orthogonal image indicative of a single droplet. We observed that the droplet had a semi-spherical shape and made contact with a bottom slide glass. **c,** Montaged images of the droplet shown in Fig. S2b (every image shows the $X-Y$ planes) at a step of $\Delta Z = 4$ µm.

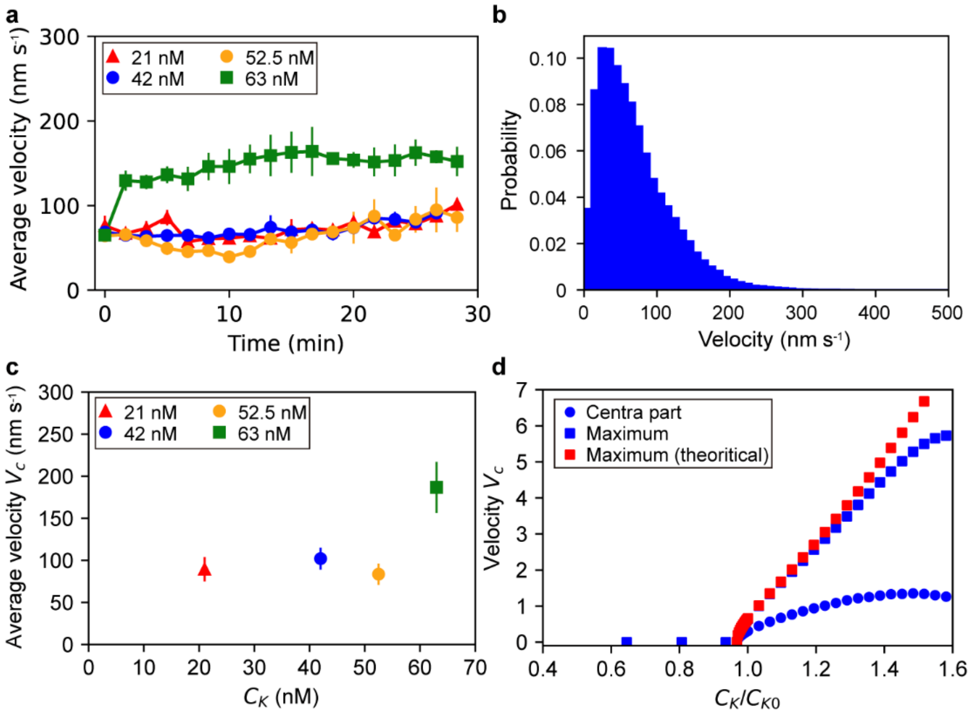

Supplementary Figure S3. Analysis of the flow velocity in the droplets. Flow velocity was assessed using the PIV analysis as shown in Fig. 2e. **a,** Time-course changes of average flow velocity at the centra part of droplets. Average values were obtained from the 3 independent droplets and error bars indicate S.D.. **b,** Distribution of the flow velocity at the concentration of kinesin, $C_K = 42$ nM, summarizing the whole values at each time point. **c,** Average flow speed depending on the kinesin concentration C_K. Each values indicate the average of $t =$ 20 - 29.5 min ($t =$ 1200 - 1770 s) shown in Fig. S3a and error bars indicate S.D.. **d,** Flow speed depending on the kinesin concentration, obtained from the numerical simulation and theoretical estimation derived from the reduced expression near the bifurcation point. Square plot indicates the maximum velocity of interface, where blue square is the result of numerical simulation, and red one is the theoretical estimation. Blue circle indicates the flow velocity of centra part obtained from numerical simulation.

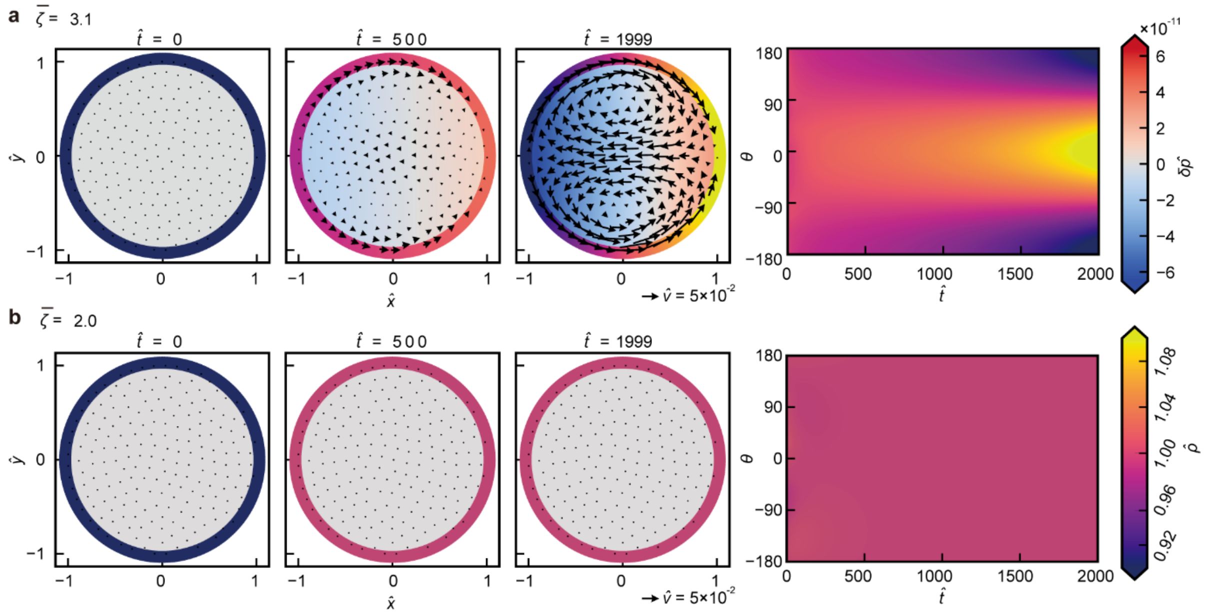

Supplementary Figure S4. Reproduced vortex flow obtained from a numerical simulation with Eqs. (1-3) with experimentally plausible values. a, Simulation result corresponds to that shown in Fig. 2e, where the spontaneous formation of a vortex was observed to be accompanied by the accumulation of ρ. **b,** Simulation result corresponds to smaller $\bar{\zeta}$; i.e., a lower kinesin concentration C_K, where both flow and localization of ρ are absent.

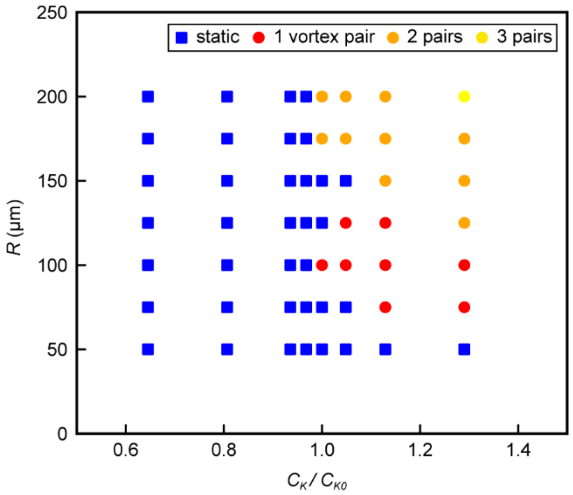

Supplementary Figure S5. Phase diagram for size R and C_K / C_{K0} based on the numerical simulation. Blue squares indicate the absence of a vortical flow. Circles indicate the presence of a vortical flow, where the number of vortex pairs is indicated by the color: red, one pair; orange, two pairs; yellow, three pairs. Typically, small R values prevent vortex formation. Larger droplets (larger R values) exhibit an increased number of vortex pairs, but the threshold $-\zeta$ may increase in size. Thus, a larger droplet does not necessarily lead to vortex formation.

Supplementary tables

Supplementary Table S1 Experimental solution for Fig. 1a

Materials	Stock concentration	Added volume	Final concentration
PEG	20%	10 μL	5%
DEX	20%	10 μL	5%
Microtubule	40 μM	1.8 μL	1.8 μM
Water	-	18.2 μL	-
	(Total volume)	40 μL	

Experimental solution for the spontaneous localization of MT inside of the DEX-rich microdroplet.

Supplementary Table S2 Experimental solution for Fig. 1b

Materials	Stock concentration	Added volume	Final concentration
PEG	20%	10 μL	5%
DEX	20%	10 μL	5%
Microtubule	40 μM	1.8 μL	1.8 μM
Kinesin	550 nM	3.1 μL	42 nM
$MgSO_4$	100 mM	4 μL	10 mM
Water	-	11.1 μL	-
	(Total volume)	40 μL	

Experimental solution for the spontaneous localization of MT/kinesin inside of the DEX-rich microdroplet.

Supplementary Table S3 Experimental solution for Figs. 1c, 2, 3, 4

Materials	Stock concentration	Added volume	Final concentration
PEG	20%	10 μL	5%
DEX	20%	10 μL	5%
Microtubule	40 μM	$C_M \cdot \frac{40}{40}$ (μL)	C_M (μM)
Kinesin	550 nM	$C_K \cdot \frac{40}{550}$ (μL)	C_K (nM)
$MgSO_4$	100 mM	4 μL	10 mM
ATP	100 mM	4 μL	10mM
Water	-	$12 - C_M - C_K \cdot \frac{4}{55}$ (μL)	-
	(Total volume)	40 μL	

Experimental solution for the spontaneous localization of MT/kinesin and formation of active vortex inside of the DEX-rich microdroplet.

Supplementary movies

Supplementary Movie S1.

Spontaneous appearance of an active vortex upon the addition of ATP, shown in Fig. 1c. C_K = 52.5 nM, C_M = 1.8 μM, and [ATP] = 10 mM. Red: MT labelled by ATTO647N, Green: Kinesin fused to eGFP, Gray: Transmitted light.

Supplementary Movie S2.

Spontaneous appearance of an active vortex upon the addition of ATP, shown in Fig. 2a, c, d. C_K = 42 nM, C_M = 1.8 μM, and [ATP] = 10 mM. Red: MT labelled by ATTO647N, Green: Kinesin fused to eGFP, Gray: Transmitted light.

Supplementary Movie S3.

Translational motion of a droplet caused by the vortical flow inside of the droplet, shown in Fig. 3. C_K = 42 nM, C_M = 1.8 μM, and [ATP] = 10 mM. Red: MT labelled by ATTO647N, Green: Kinesin fused to eGFP, Gray: Transmitted light.

Supplementary Movie S4.

Formation of a static aster-like structure upon the addition of ATP, shown in Fig. 4a, b, C_K = 42 nM, C_M = 3.6 μM, and [ATP] = 10 mM. Red: MT labelled by ATTO647N, Green: Kinesin fused to eGFP, Gray: Transmitted light.

Supplementary Movie S5.

Reproduced time-course change of a numerical simulation with Eqs. (1-3). **a,** Spontaneous formation of vortex flow shown in Fig. 2e and Supplementary Fig. S4, correspond to $\bar{\zeta} =$ 3.1. **b,** Absence of vortical flow and the localization of ρ, corresponds to $\bar{\zeta} = 2.0$.
