# Supplementary material for: Self-emergent vortex flow of microtubule and kinesin in cell-sized droplets under water/water phase separation
Source: Commun Chem. 2023 Apr 26;6:80. doi: 10.1038/s42004-023-00879-5 (PMC10133263; doi:10.1038/s42004-023-00879-5)
Supplement: Supplementary file 3 — Description of Additional Supplementary Files [file 42004_2023_879_MOESM3_ESM.pdf]

Description of Additional Supplementary Files

File name: Supplementary Movie 1

Description: Spontaneous appearance of an active vortex upon the addition of ATP, shown in Fig. 1c. $CCKK$ = 52.5 nM, $CCMM$ = 1.8 µM, and [ATP] = 10 mM. Red: MT labelled by ATTO647N, Green: Kinesin fused to eGFP, Gray: Transmitted light.

File name: Supplementary Movie 2

Description: Spontaneous appearance of an active vortex upon the addition of ATP, shown in Fig. 2a, c, d. $CCKK$ = 42 nM, $CCMM$ = 1.8 µM, and [ATP] = 10 mM. Red: MT labelled by ATTO647N, Green: Kinesin fused to eGFP, Gray: Transmitted light.

File name: Supplementary Movie 3

Description: Translational motion of a droplet caused by the vortical flow inside of the droplet, shown in Fig. 3. $CCKK$ = 42 nM, $CCMM$ = 1.8 µM, and [ATP] = 10 mM. Red: MT labelled by ATTO647N, Green: Kinesin fused to eGFP, Gray: Transmitted light.

File name: Supplementary Movie 4

Description: Formation of a static aster-like structure upon the addition of ATP, shown in Fig. 4a, b, $CCKK$ = 42 nM, $CCMM$ = 3.6 µM, and [ATP] = 10 mM. Red: MT labelled by ATTO647N, Green: Kinesin fused to eGFP, Gray: Transmitted light.

File name: Supplementary Movie 5

Description: Reproduced time-course change of a numerical simulation with Eqs. (1-3). a, Spontaneous formation of vortex flow shown in Fig. 2e and Supplementary Fig. S4, correspond to $\zeta\zeta$= 3.1. b, Absence of vortical flow and the localization of $\rho\rho$, corresponds to $\zeta\zeta$= 2.0.
