# Supplementary material for: Self-emergent vortex flow of microtubule and kinesin in cell-sized droplets under water/water phase separation
Source: Commun Chem. 2023 Apr 26;6:80. doi: 10.1038/s42004-023-00879-5 (PMC10133263; doi:10.1038/s42004-023-00879-5)
Supplement: Supplementary file 9 — Reporting Summary [file 42004_2023_879_MOESM9_ESM.pdf]

nature portfolio

Corresponding author(s): Yutaka Sumino

Last updated by author(s): Apr 6, 2023

Reporting Summary

Nature Portfolio wishes to improve the reproducibility of the work that we publish. This form provides structure for consistency and transparency in reporting. For further information on Nature Portfolio policies, see our Editorial Policies and the Editorial Policy Checklist.

Statistics

For all statistical analyses, confirm that the following items are present in the figure legend, table legend, main text, or Methods section.

n/a	Confirmed	
☐	☒	The exact sample size (*n*) for each experimental group/condition, given as a discrete number and unit of measurement
☐	☒	A statement on whether measurements were taken from distinct samples or whether the same sample was measured repeatedly
☒	☐	The statistical test(s) used AND whether they are one- or two-sided<br>*Only common tests should be described solely by name; describe more complex techniques in the Methods section.*
☒	☐	A description of all covariates tested
☒	☐	A description of any assumptions or corrections, such as tests of normality and adjustment for multiple comparisons
☐	☒	A full description of the statistical parameters including central tendency (e.g. means) or other basic estimates (e.g. regression coefficient) AND variation (e.g. standard deviation) or associated estimates of uncertainty (e.g. confidence intervals)
☒	☐	For null hypothesis testing, the test statistic (e.g. *F*, *t*, *r*) with confidence intervals, effect sizes, degrees of freedom and *P* value noted<br>*Give P values as exact values whenever suitable.*
☒	☐	For Bayesian analysis, information on the choice of priors and Markov chain Monte Carlo settings
☒	☐	For hierarchical and complex designs, identification of the appropriate level for tests and full reporting of outcomes
☒	☐	Estimates of effect sizes (e.g. Cohen's *d*, Pearson's *r*), indicating how they were calculated

Our web collection on statistics for biologists contains articles on many of the points above.

Software and code

Policy information about availability of computer code

Data collection	The confocal laser scan microscopy (Nikon A1 and Ti-E, Tokyo, Japan) was controlled by NIS-Elements C software (Nikon, Tokyo, Japan).<br>Numerical simulation was done with conventional computer with Intel(R) Core(TM) i7-5960X CPU @ 3.00GHz
Data analysis	Confocal image acquisition was performed with Nikon A1 and Ti-E (Nikon, Tokyo, Japan).<br>Segmentation and 3D reconstruction of confocal microscopic images(Fig. 1 to 4, S1 and S3) was done by NIS-Elements viewer.<br>Microscopic images (Fig. 1c, 2, 3, S1, S3) were analyzed using Image J/Fiji (version of 2021), Matlab2021b and Python 3.9.1.<br>Custom codes to conduct numerical simulation are shared on zenodo(*****)

For manuscripts utilizing custom algorithms or software that are central to the research but not yet described in published literature, software must be made available to editors and reviewers. We strongly encourage code deposition in a community repository (e.g. GitHub). See the Nature Portfolio guidelines for submitting code & software for further information.

Data

Policy information about availability of data

All manuscripts must include a data availability statement. This statement should provide the following information, where applicable:

- Accession codes, unique identifiers, or web links for publicly available datasets
- A description of any restrictions on data availability
- For clinical datasets or third party data, please ensure that the statement adheres to our policy

The data that support the findings of this study are available from the corresponding author upon reasonable request.

Human research participants

Policy information about studies involving human research participants and Sex and Gender in Research.

Reporting on sex and gender	The manuscript does not mention sex and gender, and no humans were used in this studies.
Population characteristics	No humans were used in this studies.
Recruitment	No humans were used in this studies.
Ethics oversight	No humans were used in this studies.

Note that full information on the approval of the study protocol must also be provided in the manuscript.

Field-specific reporting

Please select the one below that is the best fit for your research. If you are not sure, read the appropriate sections before making your selection.

☒ Life sciences ☐ Behavioural & social sciences ☐ Ecological, evolutionary & environmental sciences

For a reference copy of the document with all sections, see nature.com/documents/nr-reporting-summary-flat.pdf

Life sciences study design

All studies must disclose on these points even when the disclosure is negative.

Sample size	For the flow analysis in droplets, no statistical method was used to determine sample size. We found several droplets making convective flows in the same field of view that confirmed the reproducibility of our results. The data from several different samples were used, which also confirmed the reproducibility of the flow and droplet motion.
Data exclusions	No data were excluded from the analysis.
Replication	All microscopy experiments were done in at least three times for the same parameter. All attempts of replication were successful and gave similar results. Motion of droplets can be slowed down when the droplet wet the substrate.
Randomization	Any data do not require randomization process.
Blinding	Any data do not require group allocation.

Reporting for specific materials, systems and methods

We require information from authors about some types of materials, experimental systems and methods used in many studies. Here, indicate whether each material, system or method listed is relevant to your study. If you are not sure if a list item applies to your research, read the appropriate section before selecting a response.

Materials & experimental systems

n/a	Involved in the study
☒	☐ Antibodies
☒	☐ Eukaryotic cell lines
☒	☐ Palaeontology and archaeology
☒	☐ Animals and other organisms
☒	☐ Clinical data
☒	☐ Dual use research of concern

Methods

n/a	Involved in the study
☒	☐ ChIP-seq
☒	☐ Flow cytometry
☒	☐ MRI-based neuroimaging
